# Cannabidiol Modulates the Expression of Alzheimer’s Disease-Related Genes in Mesenchymal Stem Cells

**DOI:** 10.3390/ijms18010026

**Published:** 2016-12-23

**Authors:** Rosaliana Libro, Francesca Diomede, Domenico Scionti, Adriano Piattelli, Gianpaolo Grassi, Federica Pollastro, Placido Bramanti, Emanuela Mazzon, Oriana Trubiani

**Affiliations:** 1 IRCCS Centro Neurolesi “Bonino-Pulejo”, via Provinciale Palermo, Contrada Casazza, 98124 Messina, Italy; rosalianalibro@hotmail.it (R.L.); domenico.scionti@gmail.com (D.S.); bramanti.dino@gmail.com (P.B.); 2 Stem Cells and Regenerative Medicine Laboratory, Department of Medical, Oral and Biotechnological Sciences, University “G. D’Annunzio”, Chieti-Pescara, via dei Vestini, 31, 66100 Chieti, Italy; francesca.diomede@unich.it (F.D.); adriano.piattelli@unich.it (A.P.); trubiani@unich.it (O.T.); 3 Council for Research and Experimentation in Agriculture—Research Centre for Industrial Crops (CREA-CIN), 45100 Rovigo, Italy; giampaolo.grassi@gmail.com; 4 Dipartimento di Scienze del Farmaco, Universita del Piemonte Orientale, Largo Donegani 2, 28100 Novara, Italy; federica.pollastro@uniupo.it

**Keywords:** mesenchymal stem cells, cannabidiol, Alzheimer’s disease, glycogen synthase kinase 3β, amyloid beta, tau

## Abstract

Mesenchymal stem cells (MSCs) have emerged as a promising tool for the treatment of several neurodegenerative disorders, including Alzheimer’s disease (AD). The main neuropathological hallmarks of AD are senile plaques, composed of amyloid beta (Aβ), and neurofibrillary tangles, formed by hyperphosphorylated tau. However, current therapies for AD have shown limited efficacy. In this study, we evaluated whether pre-treatment with cannabidiol (CBD), at 5 μM concentration, modulated the transcriptional profile of MSCs derived from gingiva (GMSCs) in order to improve their therapeutic potential, by performing a transcriptomic analysis by the next-generation sequencing (NGS) platform. By comparing the expression profiles between GMSCs treated with CBD (CBD-GMSCs) and control GMSCs (CTR-GMSCs), we found that CBD led to the downregulation of genes linked to AD, including genes coding for the kinases responsible of tau phosphorylation and for the secretases involved in Aβ generation. In parallel, immunocytochemistry analysis has shown that CBD inhibited the expression of GSK3β, a central player in AD pathogenesis, by promoting PI3K/Akt signalling. In order to understand through which receptor CBD exerted these effects, we have performed pre-treatments with receptor antagonists for the cannabinoid receptors (SR141716A and AM630) or for the vanilloid receptor 1 (TRPVI). Here, we have proved that TRPV1 was able to mediate the modulatory effect of CBD on the PI3K/Akt/GSK3β axis. In conclusion, we have found that pre-treatment with CBD prevented the expression of proteins potentially involved in tau phosphorylation and Aβ production in GMSCs. Therefore, we suggested that GMSCs preconditioned with CBD possess a molecular profile that might be more beneficial for the treatment of AD.

## 1. Introduction

Recently, mesenchymal stem cells (MSCs) derived from gingiva (GMSCs) have gained attention as an alternative source of MSCs [1,2], because of their high availability and easier accessibility through a minimally invasive dental procedure [3]. GMSCs are multipotent cells that share a common set of surface markers with other MSCs isolated from different tissues, i.e., cluster of differentiation (CD) CD73, CD90, CD105, while they do not express CD14, CD34, CD45, and the human leukocyte antigen-DR (HLA-DR) [4]. Compared to other MSCs, GMSCs have shown a higher potential to differentiate into neural cells [5,6], likely due their neural crest embryonal origin [7], which makes them an attractive tool for the treatment of neurodegenerative diseases including Alzheimer’s disease (AD).

AD is a neurodegenerative disease characterized by two well-known pathological hallmarks: senile plaques, due to the extracellular accumulation of the amyloid beta (Aβ) protein [8], and neurofibrillary tangles (NFTs), caused by the aggregation of hyperphosphorylated tau [9]. These two insoluble protein aggregates lead to chronic inflammatory response and extensive oxidative damage, which results in progressive neuronal degeneration. Aβ is generated by the sequential cleavage of the Amyloid Precursor Protein (APP), mediated by the β-secretase (BACE-1) and γ-secretase complex [10]. The γ-secretase complex consists of at least four members: the Presenilins (PSEN1/2), Nicastrin (NCSTN), the Anterior Pharynx Defective 1 (APH-1), and the Presenilin Enhancer 2 (PSENEN). Genetic mutations in *PSEN1*, *PSEN2*, and *APP* genes have been closely linked to the familial form of AD and correlated with an increased risk to develop the sporadic form of AD [11]. Tau is a microtubule-stabilizing protein that maintains neuronal cell structure and axonal transport. During AD, tau is aberrantly phosphorylated by multiple kinases, among which the Glycogen synthase kinase-3β (GSK3β), the cyclin-dependent protein kinase-5 (CDK5), the Dual specificity tyrosine-phosphorylation-regulated kinase 1A (DYRK1A), the Calmodulin-dependent protein kinase II (CAMKII), and the Mitogen-activated protein kinases (MAPKs) are the best known [12,13,14]. To date, there are no effective therapies to counteract Aβ or p-tau formation, thus new therapeutic tools are needed.

Cannabidiol (CBD) is a non-psychotropic cannabinoid that has shown beneficial effects in AD in in vitro and in vivo models. Specifically, CBD administration in vitro inhibited the hyperphosphorylation of tau protein GSK3β-mediated in Aβ-stimulated PC12 neuronal cells [15] and reduced Aβ production in neuroblastoma cells overexpressing APP (SHSY5YAPP+) by promoting its ubiquitination [16]. Instead, in vivo CBD treatment has been shown to reverse the cognitive deficits in a double transgenic AD mouse model (APP/PS1) [17].

Numerous compounds have recently been tested for preconditioning GMSCs in vitro in order to optimize the regenerative treatment outcome in vivo [18]. In this study, we will investigate whether CBD pre-treatment in GMSCs modulated the transcription of genes involved in Aβ and tau generation. The goal of this preliminary study is to understand whether CBD may confer to GMSCs a molecular profile with better therapeutic potential for the treatment of in vivo AD models compared to control GMSCs (CTR-GMSCs).

## 2. Results

### 2.1. Morphological and Cytofluorimetric Characterization of GMSCs

**Figure 1:**
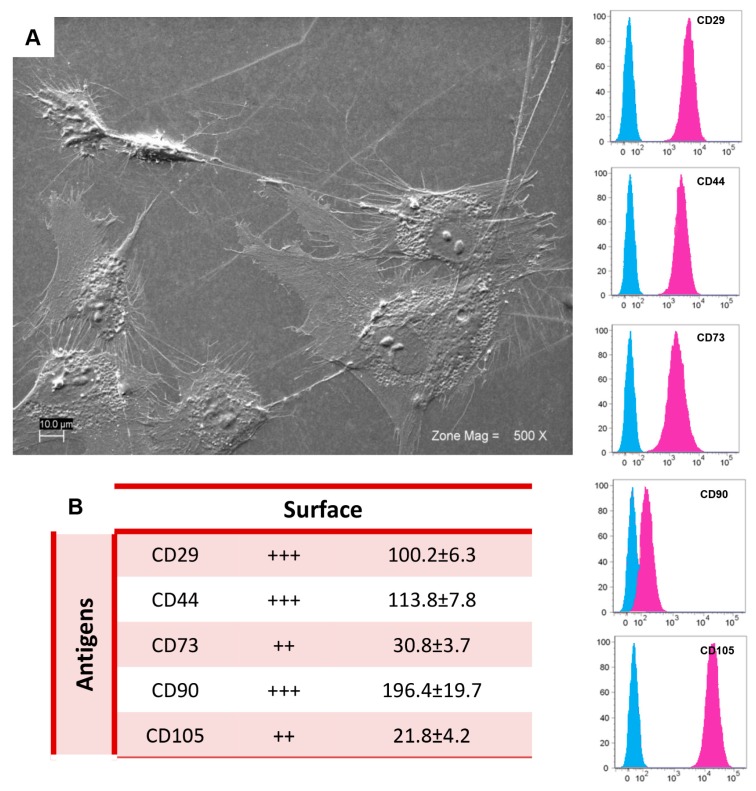
GMSCs characterization: (**A**) Ultrastructural morphology of GMSCs observed at scanning electron microscopy. Cells showed characteristic fibroblast-like morphology with several filopodia that make a contact with neighbouring cells. Magnification: 500×; (**B**) Cytofluorimetric determination of GMSCs-related antigens. The blue histogram shows the distribution of the antigen expression, whereas the pink histogram represents the respective control. The values are expressed as mean fluorescence ratio (MFI), obtained by dividing the MFI of positive events by the MFI of negative events. Numeric values of MFI ratio are the mean ± SD. ++: medium expression; +++: high expression; MFI ratio is the average of different biological samples (*n* = 5) and standard deviation.

GMSCs have shown a fibroblast-like shape, with a nucleus with one or more nucleoli (Figure 1A). In accordance with the criteria of the International Society for Cellular Therapy to define human MSCs [19], we found that primary GMSCs showed a positive expression for the cell surface antigens CD29, CD44, CD73, CD90, and CD105 (Figure 1B), while the surface molecules CD14, CD34, and HLA-DR were not expressed by these cells (data not shown).

### 2.2. CBD Treatment in GMSCs Modulated the Transcription of Genes Involved in AD Pathogenesis

**Figure 2:**
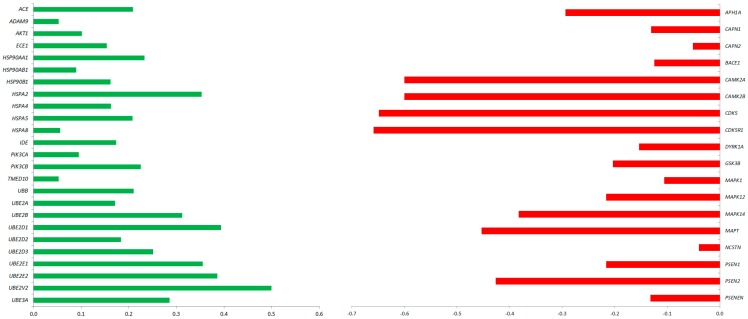
AD-related genes differentially expressed between CBD-GMSCs and CTR-GMSCs. The fold change is expressed in log10 (*q* ≤ 0.05, Benjamini–Hochberg False Discovery Rate). Upregulated genes are shown in green, while downregulated ones are in red.

In order to optimize the therapeutic potential of GMSCs, we have explored whether CBD could modulate the GMSCs transcriptional profile with regard to the genes correlated with AD pathogenesis. Specifically, gene expression analysis has that shown several genes are differentially expressed between GMSCs treated with CBD 5 μM for 24 h (CBD-GMSCs) and untreated GMSCs (CTR-GMSCs). In particular, we found that CBD treatment downregulated expression of the genes coding for kinases responsible for aberrant tau phosphorylation (*GSK3*β, *CDK5*, *DYRK1A*, *CAMK2A*, *MAPK1*, *MAPK12*, and *MAPK14*) (Figure 2; Table 1).

Moreover, the genes coding for the secretases involved in Aβ production, such as the β-(BACE-1) and γ-secretases (*PSEN1*, *PSEN2*, *NCSTN*, *PSENEN*, and *APH1A*), were found to be downregulated in CBD-GMSCs, while the genes coding for enzyme involved in Aβ degradation (*ACE1*, *ECE1*, and *IDE*) were upregulated (Figure 2; Table 2). The mRNA expression of genes coding for different heat shock proteins (HSPs), such as the HSP70s (*HSPA2*, *HSPA4*, *HSPA5*, and *HSPA8*) and the HSP90s (*HSP90AA1*, *HSP90AB1*, and *HSP90B1*), as well as the genes coding for members of the ubiquitin system and the ubiquitin-conjugating enzymes (*UBE2D1*, *UBE2D2*, *UBE2D3*, *UBE2E1*, *UBE2E2*, *UBE2V2*, and *UBE3A*) were increased in CBD-GMSCs. Moreover, we found an upregulation of the genes coding for the PI3K subunits (*PIK3CA* and *PIK3CB*) and for AKT1 in CBD-GMSCs. Conversely, in Table 1 and Table 2, apart from the AD genes differentially regulated among CTR-GMSCs and CBD-GMSCs, we have also indicated the gene ontology (GO) annotations, the gene expression levels (Exp_Value), the Fold Change (FC), and the False Discovery Rate (FDR) for each gene considered. Raw data of gene expression analysis are available in Table S1.

### 2.3. CBD Inhibited GSK3β Activity by Promoting the PI3K/AKT Signalling Pathway

**Figure 3:**
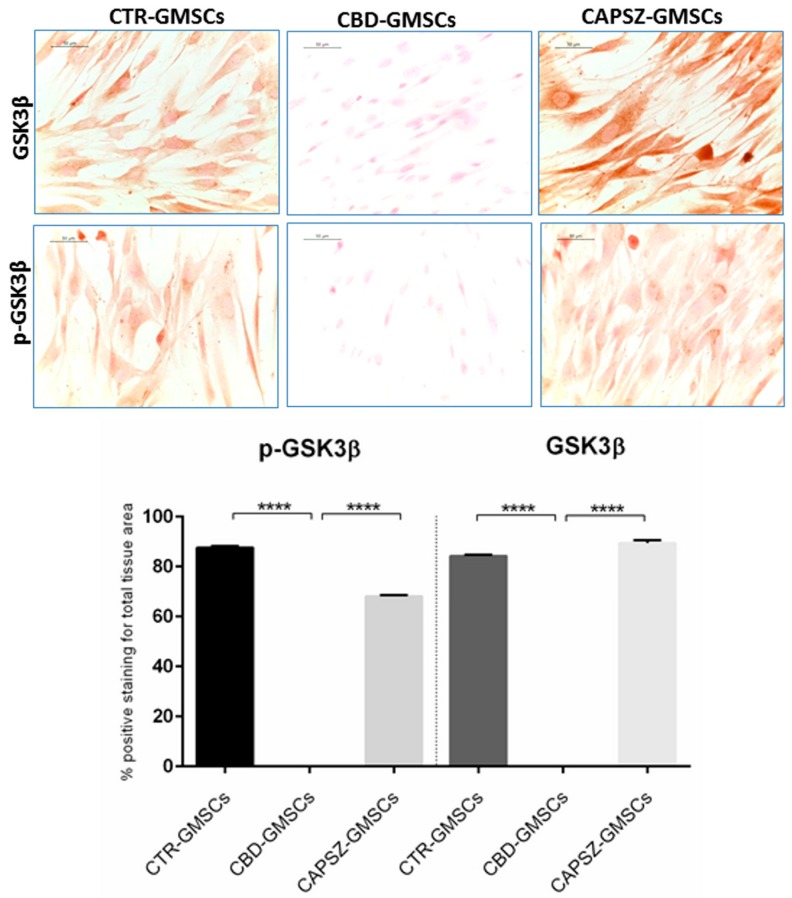
Immunostaining for GSK3β and p-GSK3β. CTR-GMSCs showed positive staining for both GSK3β and p-GSK3β, whereas CBD-GMSCs showed negative expression. GMSCs pre-treated with Capsazepine/TRPV1 antagonist (CAPSZ-GMSCs) were positive for GSK3β and p-GSK3β; CTR-GMSCs vs. CBD-GMSCs **** *p* < 0.0001; CBD-GMSCs vs. CAPSZ-GMSCs **** *p* < 0.0001. Densitometric analysis was carried out on 3/6 coverslips by covering about 90% of the total area for each experimental group. Objective: 40×, bar length: 50 μm.

The immunocytochemical results have shown a negative GSK3β and p-GSK3β staining in CBD-GMSCs (Figure 3), while CTR-GMSCs showed a positive cytoplasmatic expression for both phosphorylated (inactive) and unphosphorylated (active) protein (Figure 3). The expression of GSK3β and p-GSK3β is significantly inhibited in CBD-GMSCs compared to CTR-GMSCs (**** *p* < 0.0001).

**Figure 4:**
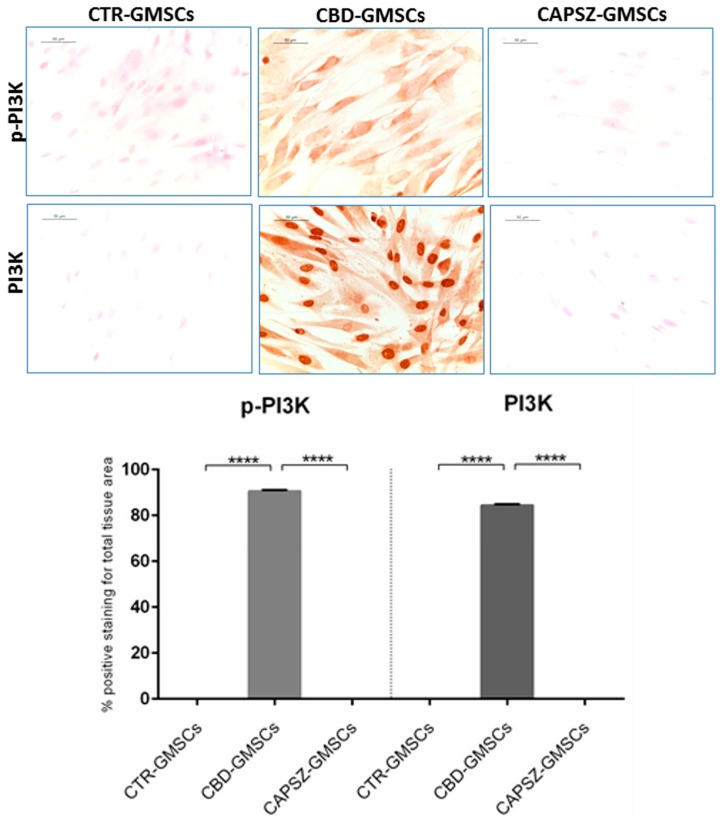
Immunostaining for p-PI3K and PI3K. CBD-GMSCs showed positive staining for p-PI3K and PI3K. On the other hand, CTR-GMSCs and showed negative expression for these proteins. The expression of p-PI3K and PI3K was abolished by pre-treatment of GMSCs with Capsazepine/TRPV1 antagonist (CAPSZ-GMSCs); CTR-GMSCs vs. CBD-GMSCs **** *p* < 0.0001; CBD-GMSCs vs. CAPSZ-GMSCs **** *p* < 0.0001. Densitometric analysis was carried out on 3/6 coverslips by covering about 90% of the total area for each experimental group. Objective: 40×, bar length: 50 μm.

**Figure 5:**
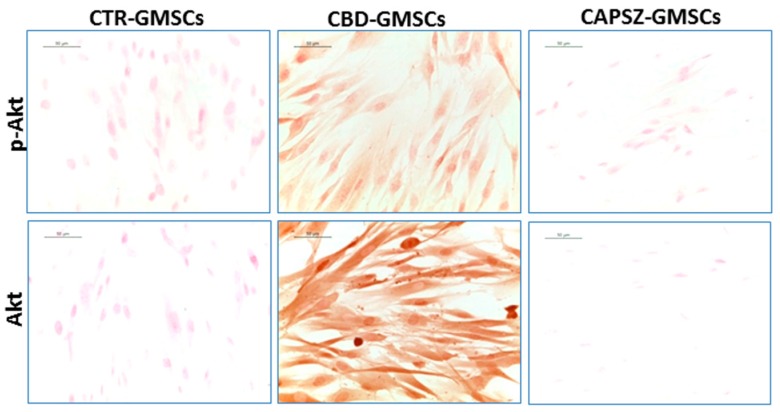

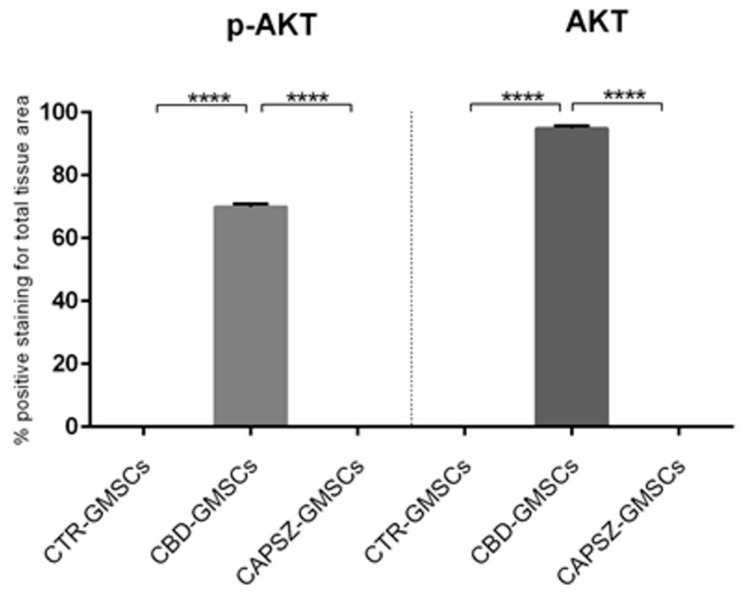
Immunostaining for p-Akt and Akt. CBD-GMSCs showed positive staining for p-Akt and Akt, whereas CTR-GMSCs and showed negative expression for these proteins. The expression of Akt and p-Akt was abolished by pre-treatment of GMSCs with Capsazepine/TRPV1 antagonist (CAPSZ-GMSCs); CTR-GMSCs vs. CBD-GMSCs **** *p* < 0.0001; CBD-GMSCs vs. CAPSZ-GMSCs **** *p* < 0.0001. Densitometric analysis was carried out on 3/6 coverslips by covering about 90% of the total area for each experimental group. Objective: 40×, bar length: 50 μm.

As regards the upstream GSK3β regulators, PI3K and AKT, we found that CBD-GMSCs showed a positive nuclear staining for PI3K, and a positive cytoplasmatic expression for p-PI3K, Akt, and p-Akt. By contrast, CTR-GMSCs showed a negative expression for these proteins (Figure 4 and Figure 5). CBD-GMSCs expressed p-PI3K, PI3K, p-Akt, and Akt in a significant manner compared to CTR-GMSCs (**** *p* < 0.0001).

Here, we have found that administration of the receptor antagonist for the transient receptor potential vanilloid 1 (Capsazepine/TRPV1) was able to antagonize the CBD-mediated effects on GSK3β, PI3K, and Akt in a significant manner (**** *p* < 0.0001) in Capsazepine-treated cells (CAPSZ-GMSCs) (Figure 3, Figure 4 and Figure 5). However, the CB1R and CB2R antagonists failed to block the increased PI3K/Akt activity induced by CBD in GMSCs, as well as the CBD-mediated inhibition of GSK3β expression (data not shown).

### 2.4. Predicted Protein Interaction Network in CBD-GMSCs

**Figure 6:**
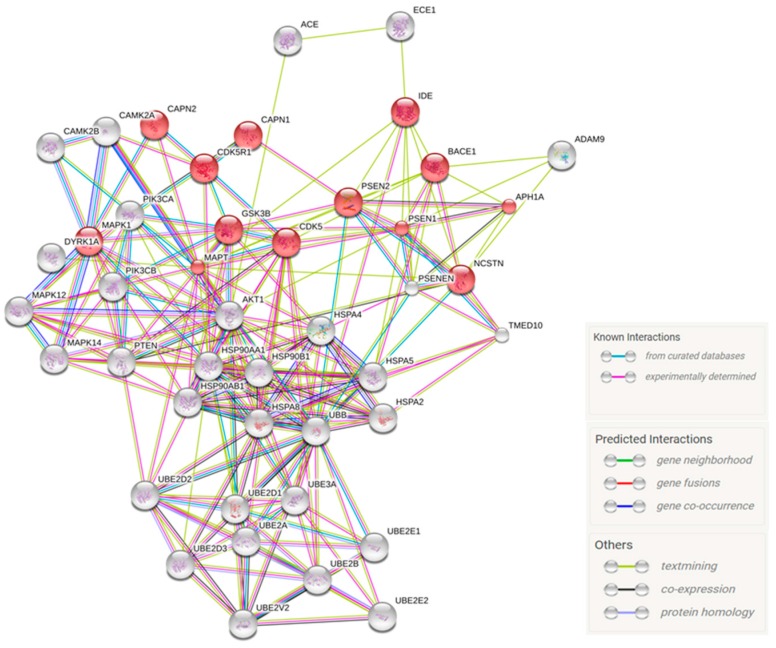
Protein association networks. The protein association network retrieved from STRING using the AD-related genes differentially modulated by CBD in GMSCs. The network circles represent proteins. The lines between the circles show the functional association. Co-expression evidence: black; database evidence: light blue; text mining evidence: yellow; experimental evidence: purple; co-occurrence evidence: blue; neighbourhood evidence: green; fusion evidence: red. The pink circles represent genes statistically correlated to the Alzheimer’s Disease pathway, according to the KEEG database, FDR = 4.19 × 10^15^.

The analysis of the genes differentially expressed among CBD-GMSCs and CTR-GMSCs was performed by inserting the selected genes on the online database STRING for pathway prediction. In this way, we obtained a putative protein association network, as showed in Figure 6.

**Figure 7:**
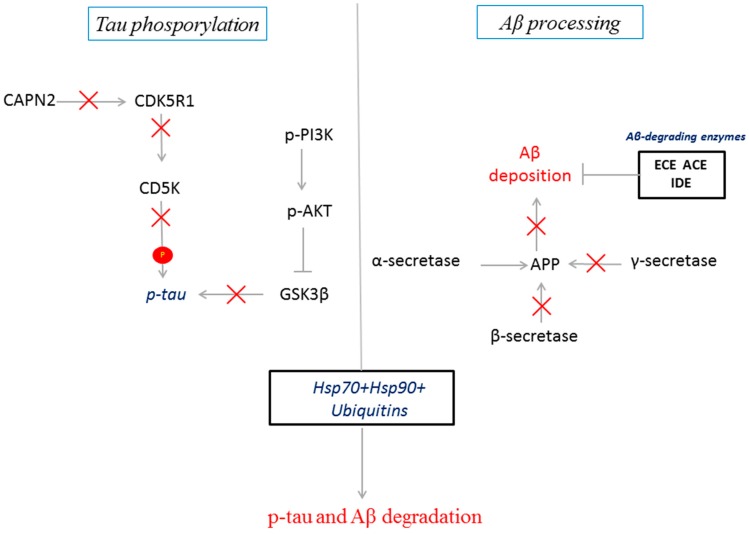
Schematic representation of the modulation of AD-related transcripts by CBD in GMSCs. CBD inhibited the transcription of the main kinases involved in tau phosphorylation (GSK3β and CDK5) and the enzymes involved in Aβ processing. In parallel, CBD potentiates the HSP70/HSP90 and the ubiquitin machineries to promote Aβ and p-tau degradation.

According to the KEEG database, *APH1A*, *BACE-1*, *CAPN1*, *CAPN2*, *CDK5*, *CDK5R1*, *DYRK1A*, *IDE*, *PSEN1*, *PSEN2*, *PSENEN*, *MAPT*, and *NCSTN* belong to the Alzheimer’s Disease pathway in a statistical manner, FDR = 4.19 × 10^15^. The putative molecular signalling modulated by CBD in GMSCs is summarized in Figure 7.

## 3. Discussion

Numerous studies have suggested a therapeutic potential for MSCs in AD. Indeed, in vivo studies have shown that MSCs can potentially regenerate damaged neuronal tissue by producing trophic factors that promote the survival and regeneration of host neurons, or alternatively can slow the progression of the disease by attenuating apoptosis, inflammation, and free radical production [2]. Specifically, Lee et al. [20] found that bone-marrow-derived MSCs infusion in the double transgenic mouse APP/PS1 reduced Aβ deposition and memory impairment by attenuating microglia activation. Similarly, MSCs derived from human umbilical cord blood, transplanted into the hippocampus of a transgenic mouse model of AD, led to significant improvement in cognitive function by reducing Aβ deposition and tau hyperphosphorylation in the brain [21]. Although MSCs transplantation in AD experimental models has been shown to exert several beneficial effects, the efficacy of this approach in clinical practice is still under evaluation through ongoing clinical trials.

Recently, MSCs derived from gingiva (GMSCs) have gained much attention as an alternative source of MSCs, because of their high availability and easy accessibility [5]. However, different studies have focused on improving the neuroprotective potential of GMSCs by preconditioning these cells with different compounds prior to their clinical application in order to improve their regenerative outcome in vivo [18]. In this study, we have evaluated whether CBD pre-treatment in GMSCs modulated the transcription of genes involved in Aβ and tau generation, in order to understand whether CBD may confer to GMSCs a molecular profile with better therapeutic potential for the treatment of in vivo AD models compared to CTR-GMSCs. The potential of CBD to influence the transcriptional profile in vitro has already been reported by Juknat et al. [22]. However, the underlying mechanisms involved in the modulatory activity of CBD have not yet been clarified.

Our results have shown that CBD pre-treatment in GMSCs modulated the transcription of a panel of genes correlated with the aetiology of AD. In particular, CBD led to the downregulation of the genes coding for the main kinases involved in tau protein phosphorylation, such as *GSK3*β [23], *CDK5* [24], *DYRK1A* [25], *CAMK2A* [26], and the *MAPKs* [27] (*MAPK1*, *MAPK12*, and *MAPK14*), indicating that CBD might prevent tau hyperphosphorylation and the consequent formation of NFTs, by reducing the transcription levels of these kinases.

In the current work, we have also found that CBD pre-treatment in GMSCs modulated the transcription of the genes involved in Aβ processing. The α-secretases promote the non-amylogenic process that drives the normal cleavage of the APP protein. Instead, the APP cleavage mediated by β-and γ-secretases is responsible for Aβ generation in AD [8,9]. Here, we find that CBD upregulated the expression of the α-secretase (*ADAM9*) and, in parallel, downregulated the β-(*BACE-1*) and γ-secretases (*PSEN1*, *PSEN2*, *NCSTN*, *PSENEN*, and *APH1A*). Additionally, the upregulation of the gene coding for negative regulator of the γ-secretase complex, *TEMED 10*, in CBD-GMSCs, correlated with decreased γ-secretase activity. We also found an upregulation of gene coding for the Aβ degrading enzymes, such as *ECE-1*, *IDE*, and *ACE*. These data might indicate that CBD prevented Aβ production by attenuating the expression of the enzymes involved in the amyloidogenic process and by promoting those implicated in the non-amyloidogenic one.

In response to exposure to Aβ peptides, the upstream activator of CDK5 kinase, CDK5R1/p35, is cleaved by CAPN2 in the p25 isoform [28], and its increased expression has been correlated with CDK5 overactivation and increased tau phosphorylation [29]. Here, we have found a downregulation of the axis CAPN2/CDK5R1 at transcriptional levels, which is consistent with the reduced CDK5 expression observed in CBD-GMSCs.

Another mechanism potentially implicated in the regulation of tau and Aβ levels could be represented by the HSPs, which are molecular chaperones involved in the maintenance of protein homeostasis, including folding, degradation, and subcellular trafficking [30]. HSPs not only recognize misfolding proteins, but also are able to promote their degradation through the ubiquitin proteasome system [31,32]; as a consequence, the upregulation of these proteins may prevent tau and Aβ misfolding and accumulation [33,34]. Our results reported an upregulated expression of different HSPs genes in CBD-GMSCs, specifically the HSP70s (*HSPA2*, *HSPA4*, *HSPA5*, and *HSPA8*) and the HSP90s (*HSP90AA1*, *HSP90AB1*, and *HSP90B1*), as well as an increased expression of the genes coding for the members of the ubiquitin system and for the ubiquitin-conjugating enzymes (*UBE2D1*, *UBE2D2*, *UBE2D3*, *UBE2E1*, *UBE2E2*, *UBE2V2*, and *UBE3A*), which indicates increased activity of the ubiquitin systems responsible for the degradation of aberrant proteins. Our data altogether indicate that CBD treatment in GMSCs negatively regulates the processing enzymes responsible for Aβ and p-tau generation and potentiates the chaperone and proteasome machineries, which promote their clearance.

Moreover, we have found an upregulation of the genes coding for the PI3K subunits (*PIK3CA* and *PIK3CB*) and for *AKT1* in CBD-GMSCs. Interestingly, PI3K/Akt signalling is known to be involved in the regulation of GSK3β activity, which by phosphorylating tau and promoting Aβ production is known to play a critical role in AD pathogenesis [35]. Immunocytochemical evaluation of PI3K/Akt/GSK3β has confirmed the results obtained through NGS analysis. These achieved data are corroborated by other authors who have highlighted the ability of cannabinoids to modulate the PI3K/Akt/GSK3β axis [36,37].

In order to understand through which receptor CBD modulated the PI3K/Akt signalling cascade, we have treated GMSCs with specific antagonists for CB1R, CB2R, or TRPV1 receptors. Notably, we have found that only the TRPV1 antagonist was able to abolish the CBD-mediated effects, indicating a direct TRPV1 involvement in this context.

TRPV1 is a calcium-permeable channel whose activation results in the elevation of intracellular calcium [38]. The intracellular concentration of calcium can influence different biological pathways, including neuronal differentiation of MSCs [39], by regulating the activity of different key enzymes.

It has been reported that CBD acting as a TRP1 agonist can activate the TRPV1 receptor in vitro, leading to its desensitization and to increased intracellular calcium levels [40]. However, the downstream molecular mechanisms activated by CBD have not yet been elucidated.

Interestingly, Hassan et al. [41] showed that CBD was able to prevent Aβ deposition in vitro, through the activation of the TRPV1 channel and thanks to its functional interaction with PI3K. However, Stein et al. [42] have demonstrated a physical association between the N-terminal region of TRPV1 and the p85β subunit of PI3K.

In accordance with this evidence, we believe that TRPV1 activation by CBD in GMSCs may trigger PI3K/Akt signalling, which in turn inactivates GSK3β through the phosphorylation at serine-9, thereby attenuating tau phosphorylation and Aβ production.

## 4. Materials and Methods

### 4.1. Extraction and Isolation of CBD

CBD was purified from an Italian variety of *Cannabis sativa* L. cultivated in the greenhouse of the Council for Research and Experimentation in Agriculture-Research Centre for Industrial Crops (CREA-CIN) in Rovigo, Italy. The purification of CBD was done in agreement with the Italian legal authorization SP/106 23/05/2013 of the Ministry of Health and it allowed us to obtain CBD pure at 99% [43].

### 4.2. Cell Culture Isolation and Characterization

GMSCs collection has been approved by the Ethics Committee for Medical School, “G. d’Annunzio” University, Chieti, Italy (n°266/17.04.14). Gingival biopsies were taken from five male volunteers without oral and systemic diseases. Sample tissues were cut into small pieces, washed using PBS (LiStarFish, Milan, Italy), and then cultured in a serum-free medium TheraPEAK™MSCGM-CD™ (Lonza, Basel, Switzerland). GMSCs migrated from gingival fragments after reaching about 80% of confluence; cells were detached by means of Triple Select (LiStarFish) [44]. GMSCs’ cytofluorimetric evaluation was performed as previously described by Diomede et al. [45]. Antibodies used for cells staining are reported in Table 3. FlowJo™ software (TreeStar, Ashland, OR, USA) was used to analyse the results obtained. Mean Fluorescence Intensity Ratio (MFI Ratio) was calculated by dividing the MFI of positive events by the MFI of negative events.

### 4.3. Scanning Electron Microscopy Analysis

GMSCs cultured on glass slices were fixed for 1 h at 4 °C in 2.5% glutaraldehyde in 0.1 M cacodylate buffer (pH 7.4); subsequently, cells were dehydrated using different ethanol concentrations until the critical point-dried is reached. Samples were gold-coated by means of an Emitech K550 (Emitech Ltd., Ashford, UK) sputter-coater and observed using scanning electron microscopy (EVO 50; Zeiss, Jena, Germany).

### 4.4. Cell Cultures and Drug Treatment

GMSCs were grown in DMEM high-glucose medium (CARLO ERBA, Milan, Italy) supplemented with 10% foetal bovine serum (FBS) (Sigma-Aldrich Co., Ltd., St. Louis, MO, USA). Cells were maintained at 37 °C in a humidified atmosphere of 5% CO_2_ and 95% air. They were cultured until they reached about 70%–80% of confluence and then divided into two experimental groups. One experimental group was obtained by pre-treating GMSCs with CBD (5 μM) for 24 h (CBD-GMSCs). The other experimental group was represented by untreated cells/control cells (CTR-GMSCs). The cytotoxicity and efficacy of the 5 μM dose of CBD were tested in our previous study [46].

In order to evaluate which receptor was involved in CBD-mediated effects, both CTR-GMSCs and CBD-GMSCs were pre-incubated for 2 h with selective receptor antagonists for cannabinoid receptor 1 (CB1R/SR141716A), for cannabinoid receptor 2 (CB2R/AM630), or for Transient receptor potential vanilloid 1 (TRPV1/Capsazepine). All receptor antagonist (Tocris Bioscience, Bristol, UK) concentrations were chosen in accordance with previous studies: specifically, the SR141716A [47,48] and Capsazepine were used at 1 μM concentration [49,50], whereas AM630 was at 100 nM [51,52].

It has been reported that GMSCs are stable cell cultures that maintain a normal karyotype and telomerase activity in long-term culture [6]. In this study, in order to obtain the largest amount of cells, GMSCs from different donors have been cultured until passage 10 and then used for the experiments.

Since we previously observed that CTR-GMSCs and CBD-GMSCs derived from different donors showed overlapping results (data not shown), here we have performed all the experiments with GMSCs derived from five donors, kept separately.

### 4.5. Preparation of RNA Libraries for Deep Sequencing

The RNA samples were extracted from CTR-GMSCs and CBD-GMSCs by using the kit RNA Cell Miniprep System (Promega, Milano, Italy). In order to obtain the RNA libraries, we have employed the TruSeq RNA Access library kit (Illumina, Inc., San Diego, CA, USA).

In brief, according to the manufacturer’s protocol, 50 ng of RNA from each sample were fragmented at 94 °C for 8 min. In a first strand phase, the cDNA were synthesized through the casual hexameres and the SuperScript II Reverse Transcriptase (Invitrogen, Milan, Italy) (incubation at 25 °C for 10 min, at 42 °C for 15 min, and at 70 °C for 15 min). In a second strand of cDNA synthesis, the RNA templates were removed and a second replacement strand was created by dUTP internalization, in order to generate double strands cDNA. The purification of the second strand reaction mix was done using AMPure XP beads (Beckman Coulter, Brea, CA, USA). The 3′ ends of the cDNA were then adenylated to allow the adaptor ligation in the following step. The AMPure XP beads were also used to purify the libraries after ligation of the indexing adaptors. An early Polymerase Chain Reaction (PCR) amplification was done to enrich those DNA fragments that have adaptors on both ends and also to increase the quantity of DNA in the library (15 cycles of 98 °C for 10 s, 60 °C for 30 s, and 72 °C for 30 s).

After library validation, a first hybridization step was done using exome capture probes. Previous hybridization of a 2-plex pool of libraries was obtained by combining 200 ng from each DNA library. The hybridization was carried out according to a standardized protocol (18 cycles, starting at 94 °C, and then decreasing by 2 °C for every cycle). In order to remove non-specific binding, we have used magnetic beads coated with streptavidin to capture probes hybridized at target regions. Another capture round with streptavidin-coated beads was performed, followed by two heated washes to discharge the non-specific binding from the beads.

The enriched libraries were then eluted from the beads and were ready for a second cycle of hybridization. This hybridization step was necessary to obtain a wide specificity of regions of capture. After, the libraries were purified through the AMPure XP bead, and amplified according to the protocol (10 cycles; incubation at 98 °C for 10 s, incubation at 60 °C for 30 s and incubation at 72 °C for 30 s), followed by a purification step.

Library quantification was done through Real-Time PCR using the KAPA Library Quantification Kit-Illumina/ABI Prism^®^ (Kapa Biosystems, Inc., Wilmington, MA, USA). Library validation was performed through a Bioanalyzer with the Agilent High Sensitivity DNA Kit (Richardson, TX, USA). Libraries were then diluted to 12 pM concentration. The single-read sequencing on the MiSeq instrument (Illumina, Inc., San Diego, CA, USA) was set at 150 cycles. The new libraries were loaded for clustering on a MiSeq Flow Cell v3 and then sequenced.

### 4.6. NGS Data Processing

Data from NGS require to be processed. First of all, sequence reads are subjected to the process of “demultiplexing” to obtain a separation of the sequence reads in different files for each index tag/sample. In order to “demultiplex” the sequence reads we have used the algorithm CASAVA (version 1.8.2, Illumina, Inc., San Diego, CA, USA). Afterwards, we did the alignment of the sequences using the RNA-Seq Alignment version 1.0.0 (Illumina, Inc., San Diego, CA, USA) and the reference sequence “Homo sapiens UCSC hg19”. For the Read mapping we used the TopHat 2 (Illumina, Inc., San Diego, CA, USA). The fragments per kilobase of exon per million fragments mapped (FPKM) values were calculated for each sample using the normalized reads counts for each annotated gene ((1000 × read count)/(number of gene covered bases × number of mapped fragments in million)). Unmapped reads were removed, preserving only the read pairs aligned to the reference sequence. The scatter plot of the LOG2 of the FPKM was used to compare two different specimens.

### 4.7. Bioinformatics Analysis

The Gene Ontology (GO) and pathway analyses of the genes differentially expressed between CTR-GMSCs and CBD-GMSCs were performed using the free tools “STRING” and “KEEG” (available online respectively at http://string-db.org and http://www.genome.jp/kegg). In this study, we only considered the GO categories or the KEGG pathways statistically significant (*q* < 0.05).

### 4.8. Immunocytochemistry

CTR-GMSCs and CBD-GMSCs were plated on coverslips of 10 mm diameter (Thermo Scientific, Oberhausen, Germany) until they reached a confluence of about 80%–90%. After that, cells were fixed with 4% paraformaldehyde for about 20 min, followed by two washes with phosphate-buffered saline (PBS, pH 7.5). In order to block the endogenous peroxidase activity, cells were exposed to 3% hydrogen peroxide (H_2_O_2_) at room temperature for 15 min. After three washes with PBS, non-specific binding sites were blocked through cell incubation with horse serum + 0.1% Triton X-100 for 20 min. Following blocking, cells were incubated overnight at 4 °C with primary antibodies against: phospho-phosphatidylinositol 3-kinase (p-PI3K, 1:100, Cell Signalling, Danvers, MA, USA), phosphatidylinositol 3-kinase (PI3K, 1:100, Cell signalling), phospho-AKT serine/threonine kinase (p-AKT, 1:100, Cell signalling), AKT serine/threonine kinase (AKT, 1:100, Cell signalling, phosphor-Glycogen synthase kinase 3β (p-GSK3β, 1:100, Santa Cruz Biotechnology, Santa Cruz, CA, USA) and Glycogen synthase kinase 3β (GSK3β, 1:100, Santa Cruz Biotechnology).

The next day, cells were incubated with a secondary antibody biotinylated (1:200, Vector Laboratories, Burlingame, CA, USA) and streptavidin ABComplex-HRP (ABC-kit from Dako, Glostrup, Denmark). The immunostaining was performed using the peroxidase substrate kit DAB (Vector Laboratories, Burlingame, CA, USA) (brown colour; positive staining), whereas the counterstaining was obtained by nuclear fast red (Vector Laboratories, Burlingame, CA, USA) (pink background; negative staining). The immunocytochemical assays were repeated three times and the experimental groups (CTR-GMSCs and CBD-GMSCs) were plated in duplicate (with a total of six coverslips for each antibody tested).

In order to calculate the percentage of positive cells stained, images were captured using light microscopy (LEICA DM 2000 combined with LEICA ICC50 HD camera) with an objective of 40×. Densitometric analysis was performed using the LEICA Application Suite V4.2.0 software (Leica Microsystems Inc., Buffalo Grove, IL, USA) on 3/6 of total coverslips, by covering about 90% of the total area for each well.

### 4.9. Statistical Analysis

In order to determine the proportion of genes differentially expressed between CTR-GMSCs and CBD-GMSCs that were statistically relevant, we have used the Cufflinks Assembly (Illumina, Inc., San Diego, CA, USA) and DE package 2.0 (Illumina, Inc., San Diego, CA, USA). The statistical evaluation of the immunocytochemistry has been carried out through the software GraphPad Prism 6.0 (GraphPad Software, La Jolla, CA, USA). The statistical relevance between CTR-GMSCs and CBD-GMSCs, as well as between CBD-GMSCs and CAPSZ-GMSCs, has been assessed by applying the one-way ANOVA statistic test and the post hoc Bonferroni test. Only *p*-values < 0.05 were considered statistically significant.

## 5. Conclusions

CBD pre-treatment in GMSCs modulated the transcriptional profile of these cells by attenuating the expression of genes implicated in the etiopathogenesis of AD. In conclusion, this preliminary in vitro study has demonstrated that GMSCs preconditioned with CBD have better therapeutic potential compared to CTR-GMSCs cells, and we believe that their transplantation in the early stage of AD may play a role in preventing or attenuating the disease onset. However, further in vivo investigations are required to assess these findings.

## Figures and Tables

**Table 1:** CBD treatment downregulated the expression of kinases involved in tau phosphorylation in GMSCs. The differential expression between CBD-GMSCs and CTR-GMSCs is given in Fold Change expressed in logarithm with base 10 (FC Log_10_). Gene ontology (GO) processes indicate the gene classification in different biological processes. Instead, the statistical significance is indicated by the False Discovery Rate (FDR), *q*-values ≤ 0.05 were considered statistically significant.

Gene	Description	GO Processes	CTR-GMSCs Exp_Value	CBD-GMSCs Exp_Value	FC Log_10_	FDR *q*-Values
*CAMK2A*	Calcium/Calmodulin Dependent Protein Kinase II α	peptidyl-serine phosphorylation, cell cycle, phosphorylation	0.02	0.00	−0.60	0.002
*CAMK2B*	Calcium/Calmodulin Dependent Protein Kinase II β	peptidyl-serine phosphorylation, protein autophosphorylation, cell cycle	0.02	0.00	−0.60	0.0082
*CAPN1*	Calpain 1	receptor catabolic process	76.18	56.41	−0.13	9.79 × 10^5^
*CAPN2*	Calpain 2	receptor catabolic process	521.29	463.30	−0.05	9.79 × 10^5^
*CDK5*	Cyclin Dependent Kinase 5	protein autophosphorylation, cell cycle, phosphorylation	12.15	2.73	−0.65	9.79 × 10^5^
*CDK5R1*	Cyclin Dependent Kinase 5 Regulatory Subunit 1	protein autophosphorylation	0.46	0.00	−0.66	9.79 × 10^5^
*DYRK1A*	Dual Specificity Tyrosine Phosphorylation Regulated Kinase 1A	peptidyl-serine phosphorylation, protein autophosphorylation, cell cycle, phosphorylation	7.34	5.15	−0.15	1.9 × 10^3^
*GSK3B*	Glycogen Synthase Kinase 3β	peptidyl-serine phosphorylation, protein autophosphorylation, cell cycle, phosphorylation	6.31	4.97	−0.20	0.005
*MAPK1*	Mitogen-Activated Protein Kinase 1	peptidyl-serine phosphorylation, cell cycle, phosphorylation	35.19	27.59	−0.11	0.002
*MAPK12*	Mitogen-Activated Protein Kinase 12	peptidyl-serine phosphorylation, cell cycle, phosphorylation	7.17	4.36	−0.72	0.0082
*MAPK14*	Mitogen-Activated Protein Kinase 14	peptidyl-serine phosphorylation, cell cycle, phosphorylation	14.07	5.83	−1.27	9.79 × 10^5^
*MAPT*	Microtubule Associated Protein Tau	cell cycle	0.34	0.00	−0.45	0.005
*APH1A*	Aph-1 Homolog A	–	17.8	9.04	−0.30	9.79 × 10^5^
*BACE1*	β-Secretase 1	β-amyloid metabolic process	7.65	5.74	−0.12	0.0011
*NCSTN*	Nicastrin	amyloid precursor protein catabolic process, apoptotic process	93.23	92.34	−0.04	0.05
*PSENEN*	Presenilin Enhancer γ-Secretase Subunit	amyloid precursor protein catabolic process, apoptotic process	69.70	51.45	−0.13	0.001
*PSEN1*	Presenilin 1	amyloid precursor protein catabolic process, apoptotic process	14.06	8.55	−0.22	9.79 × 10^5^
*PSEN2*	Presenilin 2	amyloid precursor protein catabolic process, apoptotic process	11.89	4.45	−0.43	9.79 × 10^5^

**Table 2:** CBD treatment upregulated the expression of genes linked to catabolic protein processes, response to unfolded protein and protein polyubiquitination. The differential expression between CBD-GMSCs and CTR-GMSCs is given in FC Log_10_. GO processes indicate the gene classification in different biological processes. Instead, the statistical significance is indicated by FDR, *q*-values ≤ 0.05 were considered statistically significant.

Gene	Description	GO Processes	CTR-GMSCs Exp_Value	CBD-GMSCs Exp_Value	FC Log_10_	FDR *q*-Values
*HSPA2*	Heat Shock Protein Family A (Hsp70) Member 2	positive regulation of cellular protein metabolic process, response to unfolded protein, regulation of protein modification process	4.81	10.84	0.35	9.79 × 10^5^
*HSPA4*	Heat Shock Protein Family A (Hsp70) Member 4	response to unfolded protein	67.8627	98.572	0.16	9.79 × 10^5^
*HSPA5*	Heat Shock Protein Family A (Hsp70) Member 5	response to unfolded protein, regulation of protein metabolic process, proteasome-mediated ubiquitin-dependent protein catabolic process	52.14	84.21	0.21	9.79 × 10^5^
*HSPA8*	Heat Shock Protein Family A (Hsp70) Member 8	response to unfolded protein, regulation of protein metabolic process	593.285	674.165	0.06	9.79 × 10^5^
*HSP90AA1*	Heat Shock Protein 90α Family Class A Member 1	response to unfolded protein	423.83	724.10	0.23	9.79 × 10^5^
*HSP90AB1*	Heat Shock Protein 90α Family Class B Member 1	response to unfolded protein, positive regulation of cellular protein metabolic process, regulation of protein modification process	572.907	704.588	0.08	9.79 × 10^5^
*HSP90B1*	Heat Shock Protein 90β Family Member 1	response to unfolded protein, proteolysis, proteasome-mediated ubiquitin-dependent protein catabolic process	262.43	380.62	0.16	9.79 × 10^5^
*UBB*	Ubiquitin B	proteolysis, protein polyubiquitination, protein catabolic process, proteasome-mediated ubiquitin-dependent protein catabolic process	2458.27	3992.14	0.21	9.79 × 10^5^
*UBE2A*	Ubiquitin Conjugating Enzyme E2 A	proteolysis, protein polyubiquitination, protein catabolic process, proteasome-mediated ubiquitin-dependent protein catabolic process	21.79	32.28	0.17	9.79 × 10^5^
*UBE2B*	Ubiquitin Conjugating Enzyme E2 B	proteolysis, protein polyubiquitination, protein catabolic process, proteasome-mediated ubiquitin-dependent protein catabolic process	15.83	32.47	0.31	9.79 × 10^5^
*UBE2D1*	Ubiquitin Conjugating Enzyme E2 D1	proteolysis, protein polyubiquitination, protein catabolic process, proteasome-mediated ubiquitin-dependent protein catabolic process	2.71	6.7154	0.39	9.79 × 10^5^
*UBE2D2*	Ubiquitin Conjugating Enzyme E2 D2	proteolysis, protein polyubiquitination, protein catabolic process, proteasome-mediated ubiquitin-dependent protein catabolic process	11.55	17.63	0.18	9.79 × 10^5^
*UBE2D3*	Ubiquitin Conjugating Enzyme E2 D3	proteolysis, protein polyubiquitination, protein catabolic process, proteasome-mediated ubiquitin-dependent protein catabolic process	21.63	38.56	0.25	9.79 × 10^5^
*UBE2E1*	Ubiquitin Conjugating Enzyme E2 E1	proteolysis, protein polyubiquitination, protein catabolic process	26.54	60.20	0.36	9.79 × 10^5^
*UBE2E2*	Ubiquitin Conjugating Enzyme E2 E2	proteolysis, protein polyubiquitination, protein catabolic process	8.10	19.69	0.39	9.79 × 10^5^
*UBE2V2*	Ubiquitin Conjugating Enzyme E2 V2	proteolysis, protein polyubiquitination, protein catabolic process	8.10	19.70	0.39	9.79 × 10^5^
*UBE3A*	Ubiquitin Protein Ligase E3A	proteolysis, protein polyubiquitination, protein catabolic process	21.35	41.18	0.29	9.79 × 10^5^
*ACE1*	angiotensin I converting enzyme	β-amyloid metabolic process	8.88	14.37	0.21	9.79 × 10^5^
*ECE1*	endothelin converting enzyme 1	β-amyloid metabolic process	34.80	49.57	0.15	9.79 × 10^5^
*IDE*	insulin degrading enzyme	β-amyloid metabolic process	9.41	14.047	0.17	9.79 × 10^5^
*ADAM9*	ADAM metallopeptidase domain 9	regulation of cellular catabolic process	324.75	366.63	0.05	9.79 × 10^5^
*PIK3CA*	phosphatidylinositol-4,5-bisphosphate 3-kinase catalytic subunit α	regulation of protein metabolic process	14.76	18.38	0.10	9.79 × 10^5^
*PIK3CB*	phosphatidylinositol-4,5-bisphosphate 3-kinase catalytic subunit β	regulation of protein metabolic process	3.33	5.59	0.22	9.79 × 10^5^
*AKT1*	AKT serine/threonine kinase 1	regulation of protein metabolic process, positive regulation of biological process, protein phosphorylation	47.16	59.5	0.10	9.79 × 10^5^
*TMED10*	transmembrane p24 trafficking protein 10	–	40.2452	45.459	0.05	0.002

**Table 3:** Antibodies and concentrations used for cell characterization.

Antibody	Manufacturer	Concentration
CD44-FITC	Ancell (Bayport, MN, USA)	1:50
CD29-PE		
CD105-FITC		
CD14-FITC	Miltenyi Biotec (Bergisch Gladbach, Germany)	
HLA-DR-PE	Becton Dickinson (San Jose, CA, USA)	
CD90-FITC		
CD73-PE		
CD34-PE	Beckman Coulter (Brea, CA, USA)	

Fluorescein isothiocyanate (FITC); Phycoerythrin (PE).

## Supplementary Materials

Supplementary materials can be found at www.mdpi.com/1422-0067/18/1/26/s1.

## Abbreviations

AD	Alzheimer’s Disease
Aβ	Amyloid Beta
CBD	Cannabidiol
CD	Cluster of Differentiation
FPKM	Fragments per Kilobase of Exon per Million Fragments Mapped
GMSCs	Gingiva Mesenchymal Stem Cells
GO	Gene Ontology
GSK3β	Glycogen Synthase Kinase 3β
HLA	Human Leukocyte Antigen-DR
HSPs	Heat Shock Proteins
MSCs	Mesenchymal Stem Cells
